# Exposure to Fungal Volatiles Can Influence Volatile Emissions From Other Ophiostomatoid Fungi

**DOI:** 10.3389/fmicb.2020.567462

**Published:** 2020-09-17

**Authors:** Fuai Wang, Jonathan A. Cale, Altaf Hussain, Nadir Erbilgin

**Affiliations:** Department of Renewable Resources, University of Alberta, Edmonton, AB, Canada

**Keywords:** fungal chemical ecology, insect-fungus interactions, bark beetles, mountain pine beetle, pine engraver beetle

## Abstract

Fungal volatile organic compounds (FVOCs) can act as intra- and inter-kingdom communication signals that influence the growth and behaviors of organisms involved in antagonistic or mutualistic relationships with fungi. There is growing evidence suggesting that FVOCs can mediate interactions between organisms within and across different ecological niches. Bark beetles have established mutualistic relationships with ophiostomatoid fungi which can serve as a food source and condition host plant tissues for developing beetle larvae. While the profiles (both composition and concentrations) of volatile emission from ophiostomatoid fungi can be influenced by abiotic factors, whether emissions from a given fungal species can be influenced by those from another is still unknown. Here, we analyzed FVOCs emitted from the two ophiostomatoid fungi, *Grosmannia clavigera* and *Ophiostoma ips*, associated with mountain pine beetle and pine engraver beetle, respectively, when each fungus was growing alone or in a shared headspace. We used two isolates of each fungus species. Overall, we detected a total of eight volatiles in both *G. clavigera* alone or in combination with *O. ips* including acetoin, ethyl acetate, *cis*-grandisol, isoamyl alcohol, isobutanol, 2-methyl-1-butanol, phenethyl acetate, and phenethyl alcohol. The profiles of volatiles emitted differed between the two fungal species but not between the two isolates of the same fungus. Six compounds were common between the species, whereas two compounds were detected only when *G. clavigera* was present. Moreover, the majority of volatiles were detected less frequently and at lower concentrations when the two fungi were grown together in a shared headspace. These results are likely due to reduced volatile emissions from *O. ips* in the presence of *G. clavigera*. However, changes in the profiles of fungal volatiles did not correspond with the observed changes in the growth of either species. Overall, these results suggest that the similarities in fungal volatiles among different species of fungi may reflect a common ecological niche and that the differences may correspond to species-specific adaptation to their respective host beetles or genetic factors.

## Introduction

Ecological interactions between fungi and other organisms can be mediated by fungal volatile organic compounds (FVOCs) (Davis et al., 2013; Hung et al., 2015; Kandasamy et al., 2016, 2019; Schulz-Bohm et al., 2017). These compounds can act as intra- and inter-kingdom communication signals to influence the behaviors of animals involved in antagonisms or mutualisms with fungi (Schulz-Bohm et al., 2017; Schenkel et al., 2018; Zhao et al., 2019). Intra- and inter-specific interactions between fungi can also be mediated by FVOCs (Hofstetter et al., 2005; Cale et al., 2016; El Ariebi et al., 2016; Wang et al., 2020). For example, volatiles of *Penicillium paneum* can inhibit spore germination of conspecifics and other species of fungi, representing a variety of genera (Chitarra et al., 2004). Moreover, VOCs emitted by older cultures of some fungal species can stimulate or inhibit the growth and reproduction of cultures of other species (Cale et al., 2016). How the outcomes of such interactions could be indirectly influenced by factors that affect FVOC emissions is unknown.

Many factors affect the profiles of FVOCs (both composition and concentrations of individual compounds) emitted by a fungus (Hung et al., 2015). Fungal VOCs can be produced directly and indirectly from metabolic processes in fungal cells (Martín et al., 2014); thus, genetic factors likely underlie some of the intra- and inter-specific variation in FVOC emissions (Polizzi et al., 2012; Buśko et al., 2014). Emissions also can be influenced by the environment in which a fungus is growing. For instance, large variations in FVOCs can result from apparent interactions among abiotic factors such as temperature, humidity, and growth substrate (Polizzi et al., 2012). Likewise, VOCs originating from host plants and insects can influence emission of FVOCs (Cale et al., 2019). However, how ecological factors may affect the profiles of FVOC emitted is unknown. Understanding the semiochemical aspects of fungal ecology can help clarify the mechanisms underlying interactions between co-occurring species that occupy similar niches. This is especially important for understanding the ecology of economically important fungi, such as the species of ophiostomatoid fungi (Ophiostomataceae and Ceratocystidaceae) vectored by tree-killing bark beetle species (Coleoptera: Curculionidae, Scolytinae).

Several species of bark beetles have caused extensive forest mortality throughout the world (Seidl et al., 2008; Bentz et al., 2010). This mortality is in part due to infections of tree vascular tissues by mutualistic ophiostomatoid fungi vectored by the beetles (Six, 2013). The fungi can support beetles by serving as a nutritional supplement (i.e., nitrogen) and by conditioning tree tissue for developing larvae (Bleiker and Six, 2007; Six, 2013; Therrien et al., 2015; Ojeda-Alayon et al., 2017). The successful colonization of host trees by beetles can also be supported by the use of complex semiochemical systems derived from host tree, beetle and fungal cues, that help beetles coordinate the activities of conspecifics in overwhelming healthy trees that otherwise resist attack (Erbilgin, 2019). These semiochemical systems could potentially be supported in part by beetle mutualistic fungi, which can emit a wide array of FVOCs including some that influence beetle behavior (Hanssen, 1993; Hofstetter et al., 2005; Cale et al., 2016, 2019; Kandasamy et al., 2016; Zhao et al., 2019). Because multiple ophiostomatoid species often co-occur in a given tree (Roe et al., 2011), FVOC-mediated interactions may potentially occur among co-occurring fungi in nature. While the FVOCs from cultures of some fungi can inhibit the growth and reproduction of other fungal species (Hofstetter et al., 2005; Cale et al., 2016; Kandasamy et al., 2019), whether these effects are associated with changes to the profiles of FVOCs emitted by the recipient fungus is unknown. However, understanding such changes could help elucidate novel aspects of the ecology of bark beetle-ophiostomatoid fungus symbioses.

Large-scale outbreaks of the mountain pine beetle MPB, *Dendroctonus ponderosae* Hopkins have killed millions of hectares of mostly lodgepole pine (*Pinus contorta* Douglas var. latifolia Engelmann) forests in western North America (Bentz et al., 2010; Safranyik et al., 2010). Moreover, warmer temperatures have allowed MPB to expand their range eastward from lodgepole pine-dominated forests into jack pine (*Pinus banksiana* Lambert)-dominated forests (Cullingham et al., 2011; Erbilgin et al., 2014) where it is likely interact with another species of bark beetle, the pine engraver beetle (*Ips pini* Say) (Kegley et al., 1997). Co-occurrence of these two beetle species in lodgepole pine can negatively affect the number of emerging beetles of either species (Rankin and Borden, 1991), which may in part result from interactions between the ophiostomatoid fungal species commonly vectored by each of these beetle species. How interactions between *Grosmannia clavigera* (Robinson-Jeffrey and Davidson) Zipfel, de Beer, and Wing, the primary fungus vectored by MPB (Lee et al., 2005, 2006), and *Ophiostoma ips* (Schenk and Benjamin, 1969; Kopper et al., 2004), the primary fungus vectored by *I. pini*, influence either fungus is poorly understood. While variation in host plant substrate quality (e.g., amounts of host defense compounds and nutrients) can influence how interactions between *G. clavigera* and *O. ips* influence the growth of either fungus (Wang et al., 2020), how such interactions could affect FVOC production or be mediated by FVOCs is unknown. Clarifying such aspects of *G. clavigera*–*O. ips* interactions is important to understanding the development of the fungi in the jack pine trees and, thus, understanding the success and persistence of their associated host bark beetles in the conifer forests of the Western North America.

Here, we collected FVOCs from and conducted laboratory bioassays with two fungal species, *G. clavigera* and *O. ips*. Specifically, headspace FVOCs above separate cultures of *G. clavigera* and *O. ips* were identified, quantified, and compared among treatments where cultures occurred alone or in combination in a shared headspace, and tested for potential effects of FVOCs on fungal growth. This approach was used to address two related research questions: (i) does the quality and/or quantity of FVOCs emitted from an ophiostomatoid fungus change when the fungus is growing in the presence of FVOCs from a different ophiostomatoid species?, and (ii) does the presence of FVOC emissions affect the growth of another ophiostomatoid fungi?

## Materials and Methods

### Fungal Growth and Volatile Collections

The following experiments used the two isolates each for *G. clavigera* and *O. ips* that were reported in Cale et al. (2019) and Wang et al. (2020). For *G. clavigera*, both isolates (NOF2894 and NOF2896) were provided by the Northern Forestry Centre Culture Collection (Edmonton, Alberta) and were originally cultured from the phloem of MPB-infested lodgepole pine trees near Banff, Alberta (Rice et al., 2007). The *O. ips* isolates (NOF1205 and NOF1284) were isolated from bark beetle galleries in lodgepole pine (Cale et al., 2019). Master cultures of the four isolates were grown on potato dextrose agar (PDA) at 22°C in total darkness for 10 days. These cultures were subcultured onto PDA media in small Petri plates (60 mm diam.) at 22°C in total darkness for 4 days. These subcultures were used in the experiment described below. Throughout the paper, isolate refers different isolates of the same fungal species while species refer to the two fungal species (*G. clavigera* and *O. ips*).

Two-day old cultures of fungi were used in the following three treatment groups for the collection of headscape volatiles by air entrainment: (1) a culture of *G. clavigera* (Gc Control), (2) a culture of *O. ips* (Oi Control), and (3) separate cultures of *G. clavigera* and *O. ips* together (Combination). For the fungus-control treatments, a single 2-day old culture plate of the respective fungus was closed inside a volatile collection apparatus (see details under Fungal volatile collection apparatus subheading below). For the combination treatment, one culture plate from one species of fungus (either *G. clavigera* or *O. ips*) was placed 5–6 cm below a culture of the other fungus in the same chamber. The higher culture plate was held in place by a wire support. We randomly determined the placement of the fungus in the volatile collection apparatus to remove any bias. Since the air inside the apparatus was circulated, both fungi were likely equally affected by one another. Furthermore, Cale et al. (2016) showed that the vertical placement of fungal cultures inside the collection apparatus did not significantly affect fungal growth of volatile emissions. Lids of the culture plates were moved to cover only half of the plate area. Headspace volatiles from cultures in the control and combination treatments were then sampled for 48 h. Cultures of the two control treatments were each replicated ten times (*N* = 5 for each isolate). The combination treatment was replicated 12 times, representing three replicates from each of four isolate combinations (*N* = 3 for each isolate). Headspace volatiles were also collected from non-inoculated PDA plates (*N* = 10; PDA control) in order to help distinguish compounds emitted by fungi from those emitted by the media (data not shown); these compounds were removed from analysis. Total fungal growth (mm^2^) on each culture plate was measured by image analysis using ImageJ software (Abramoff et al., 2004). We used the same sampling apparatus to sample volatiles from each group of four treatments including the control. The mean (±SE) 4-day growth was calculated for cultures of each isolate (1845.7 ± 142.2 mm^2^ for NOF2894, 1905.0 ± 73.6 mm^2^ for NOF2969, 1397.7 ± 49.4 mm^2^ for NOF1284, and 1095.7 ± 28.3 mm^2^ for NOF1205).

### Fungal Volatile Collection Apparatus

The volatile collection apparatus was similar to one reported in Cale et al. (2016) (Supplementary Figure S1). This was a closed-air system consisting of a 473 mL glass jar (collection chamber) whose threading was wrapped with Teflon tape and fitted with a metal cap fitted with two pairs of brass spigots (each pair fitted together by brass Swagelok) such that each pair consisted of a spigot extending above and below the cap surface. These spigots served as channels to allow constant airflow (475 mL min^–1^) using a flowmeter into and out of the collection chamber. The inlet channel was attached to 30.5 cm piece of Teflon tubing packed halfway down with activated carbon (800 mg; 6–14 mesh; held in place with glass wool) in order to purify air entering the collection chamber (air scrubber). This tubing was attached to a stainless-steel gang-valve connected to the outlet spigot of a bellows vacuum/pressure pump. A 15 cm section of Teflon tubing was attached to the outlet channel and attached to an adsorbant trap consisting of a 7.5 cm piece of Teflon tubing packed with 150 mg of activated carbon (held in place with glass wool). This trap collected culture headspace volatiles carried in the air stream as it flowed out of the collection chamber. The trap was attached to an eight cm long tube that joined the outlet channel to a gang-valve connected to the inlet spigot of the pump. A tight wrapping of Teflon tape was used at sites of tube and fitting connections in order to prevent outside air from entering and, thus, contaminating the system. The two gang-valves consisted of four spigots each, allowing us to connect to a set of four collection chambers/systems to each pump. Sample sets consisted of one replicate from each treatment plus a PDA control.

The sampling of headspace volatiles occurred over 48 h after culture/control plates were sealed in the collection chambers. The first 24 h represented a “charge period” that allowed headspace volatiles to accumulate in the jars before air started flowing through the system. The charge period was immediately followed by a 24 h “collection period” when the pumps were engaged and volatiles carried in an airstream were collected in the adsorbent traps. Pumps were then disengaged at the end of the collection period, and trap tubes were detached from the system, wrapped in labeled aluminum foil, and stored at −40°C prior to chemical extraction. Collection jars and caps were cleaned with acetone prior to each sampling set.

### Chemical Analysis of FVOCs

Fungal VOCs were extracted from collection traps by placing the adsorbant into a 2 mL tube containing 1 mL of dichloromethane containing a tridecane as an internal standard (0.001%). The mixture was vortexed for 30 s, sonicated for 10 min, and centrifuged (18,213 rcf) at 0°C for 30 min. The extract was collected and transferred to a 2 mL gas chromatograph (GC) vial. This procedure was repeated a second time. The extracts were then analyzed using a GC (Agilent Tech., Santa Clara, CA, United States) fitted with a DB-5MS column (30 m length, 0.25 μm film, 0.25 mm I.D.; Agilent Tech., Santa Clara, CA, United States) and coupled to a mass spectrometer (GC-MS; GC: 7890A, MS: 5975C; Agilent Tech.). Helium was used as a carrier gas flowing at 1 mL min^–1^ with a temperature program beginning 50°C (held for 1 min) then increased by 5°C min^–1^ to 200°C, followed by an increase of 30°C min^–1^ to 325°C (held for 2 min). A 1 μL sample injection was used, and samples were run in splitless mode. Peaks present in the PDA controls were ignored when analyzing samples from the treatments. Peak identifications were confirmed using a NIST/EPA/NIH Mass Spectral library version 2.0f and analytical standards. The following standards were used: acetoin (≥96% chemical purity), ethyl acetate (≥99%), *cis*-grandisol [(1R,2S)-*cis*-2-isopropenyl-1-methylcyclobutaneethanol; ≥96%], isoamyl alcohol (≥98%), isobutanol (≥99%), 2-methyl-1-butanol (≥99%), phenethyl acetate (≥98%), and phenethyl alcohol (≥99%). All standards were purchased from Sigma-Aldrich (St. Louis, MO, United States) except *cis*-gransisol, which was purchased from Alpha Scents (West Linn, OR, United States). We quantified each individual compound using standard curves calculated from three serial dilutions of analytical standards. The internal standard (tridecane) was used to improve the precision of quantitative analysis with the calibration curve by plotting the signal from analyte with the signal from the internal standard as a function of the analyte concentration of the standards. The concentrations of the headspace volatiles detected were calculated as the amount of compound per unit culture area (as mean area of 4-day growth for a given isolate) per day (ng/mm^2^/day).

### Fungal Growth Bioassays

The bioassays of fungal growth responses to FVOCs used new subcultures (on 60 mm PDA plates) of the same *G. clavigera* and *O. ips* isolates from which FVOCs were collected above. Bioassays were overall designed to expose a newly inoculated subculture (response culture) of a fungus to FVOCs emitted from 2- to 4-day old cultures (source cultures). The three plates were sealed together inside a glass jar (473 mL) such that the source culture plates were adjacent to each other on the bottom of the jar and the response culture was held 5–6 cm above them on a support of coiled wire. This approach was used to test the effects of source treatments on the growth of either *G. clavigera* or *O. ips*. Four source treatments were used: two plates of *G. clavigera*, two plates of *O. ips* one plate of each species of fungus (combination treatment), or two non-inoculated PDA plates as control. Isolates from each species of the fungus were randomly selected. The eight treatments were replicated ten times, with each isolate of a given fungus representing half of the replicates. Response cultures were grown in this manner for either three (*G. clavigera*) or four (*O. ips*) days to account for inherent inter-specific differences in growth rates. The total area (mm^2^) of response cultures was measured by image analysis using ImageJ software at this time (Abramoff et al., 2004).

### Data Analysis

To test difference in the qualitative variation of fungal headspace VOC profiles (both composition and concentration of individual compounds) for statistical significance among collection treatments, matrices of the presence/absence of individual compounds (detection profiles) were constructed for each sample and analyzed using two-way permutational multivariate analysis of variance (PERMANOVA). This PERMANOVA tested the significance of main effects of isolate and treatment along with an effect of isolate-treatment interaction. Significant effects on the quality profiles were visualized using principle coordinate analysis (PCoA). To analyze differences in the quantitative variation of these VOCs, total VOC concentrations were calculated by summing the concentrations of each individual compound detected in a given sample. Total concentration differences among collection treatments were tested for statistical significance using two-way analysis of variance (ANOVA), which tested main effects of isolate and treatment and an isolate-treatment interaction effect. Pairwise Tukey Honest Significant Difference (HSD) tests were conducted following the identification of significant effects. Quantitative differences among collection treatments were also analyzed using profiles of individual compound concentrations. Two-way PERMANOVA was used to test statistical significance of isolate and treatment main effects as well as an isolate-treatment interaction, with significant effects being visualized using PCoA.

Fungal growth responses to VOC source treatments were analyzed separately for *G. clavigera* and *O. ips* response cultures. For each species, two-way ANOVA was used to test the statistical significance of source treatment and the isolate main effects of the responding fungus. Treatment-isolate interactions were also tested for significance. Pairwise comparisons were performed using Tukey HSD tests for significant effects.

Data were log-transformed to satisfy assumptions of normality and heteroscedasticity of the ANOVAs, as needed. All data analyses were conducted in the R software environment version 3.6.0 (R Core Team, 2019). All PERMANOVAs and PCoAs were calculated and tested using functions provided by the R package “vegan” version 2.5-5 (Oksanen et al., 2019).

## Results

### Fungal Volatile Responses

**FIGURE 1:**
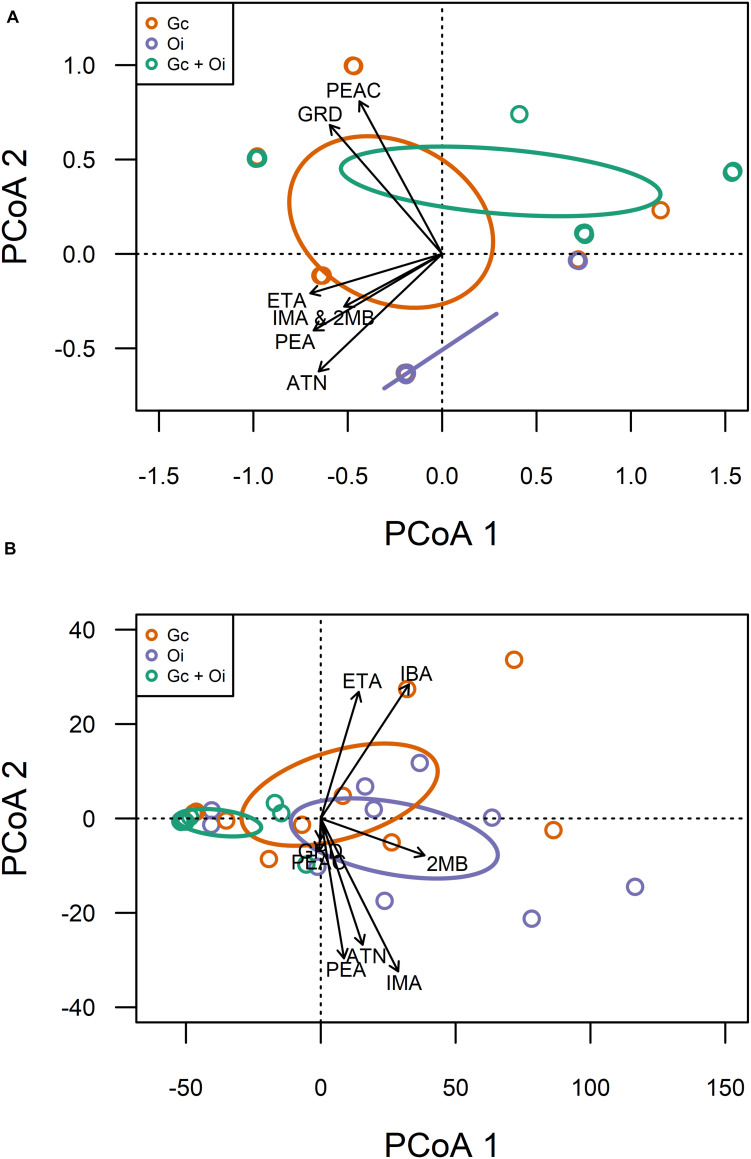
Qualitative **(A)** and quantitative **(B)** variation in volatile organic compound profiles detected from the headspace of *Grosmannia clavigera* (Gc; orange) cultures, *Ophiostoma ips* (Oi; purple) cultures, and these fungi together (Gc + Oi; green). Circles represent 95% confidence ellipses around cluster centroids. Acronyms for chemicals: ATN, Acetoin; IBA, Isobutanol; 2MB, 2-methyl-1-butanol; IMA, Isoamyl alcohol; PEA, Phenethyl alcohol; GRD, *cis*-Grandisol; ETA, Ethyl acetate; PEAC, Phenethyl acetate.

Eight compounds were detected from the headspace of *G. clavigera* and *O. ips* isolates growing either alone (fungal controls) or in the presence of the other species (combination): acetoin, ethyl acetate, *cis*-grandisol, isoamyl alcohol, isobutanol, 2-methyl-1-butanol, phenethyl acetate, and phenethyl alcohol (Table 1). However, the FVOC profiles of compounds varied among treatments, as the profiles were influenced by fungal species and whether the fungus was growing alone or in the presence of FVOCs of the other species. The FVOC profiles differed among the three treatments (PERMANOVA *F*_(2,26)_ = 3.54, *P* = 0.003; Figure 1A), with the combination treatment having profiles similar to that of the *G. clavigera* control but different from the *O. ips* control. Profiles from these controls differed from each other. However, the fungal isolates (or isolate combinations) used did not influence FVOC emission as the isolate main effect and the isolate-treatment interaction effect were not significant. The inter-treatment patterns in FVOC profiles were likely due to variation in the total number of compounds detected and their detection frequencies (percentage of samples) among the treatments (Table 1). Similarly, all compounds detected from the combination treatment were detected at least once from either of the fungal controls, indicating that for a given fungus, the presence of the other fungus did not elicit emission of any novel compounds (Table 1). However, *cis*-grandisol and phenethyl acetate were not detected from the *O. ips* controls. The detection frequencies of all compounds other than isobutanol were lowest for the combination treatment (Table 1).

**TABLE 1:** Percentage of samples in which individual volatile organic compounds were detected from headspace collections of *Grosmannia clavigera* (*N* = 10), *Ophiostoma ips* (*N* = 10), and *G. clavigera* plus *O. ips* grown on potato dextrose agar (*N* = 9).

	Percentage of samples compound was detected		
	
Compounds	*Grosmannia clavigera*	*Ophiostoma ips*	*G. clavigera* + *O. ips*
Acetoin	60	80	33
Ethyl acetate	80	80	78
*cis*-Grandisol	60	ND	33
Isoamyl alcohol	100	100	78
Isobutanol	100	100	100
2-Methyl-1-butanol	100	100	78
Phenethyl acetate	40	ND	44
Phenethyl alcohol	90	100	33

*Compounds not detected in a given collection group are indicated with “ND.”*

**FIGURE 2:**
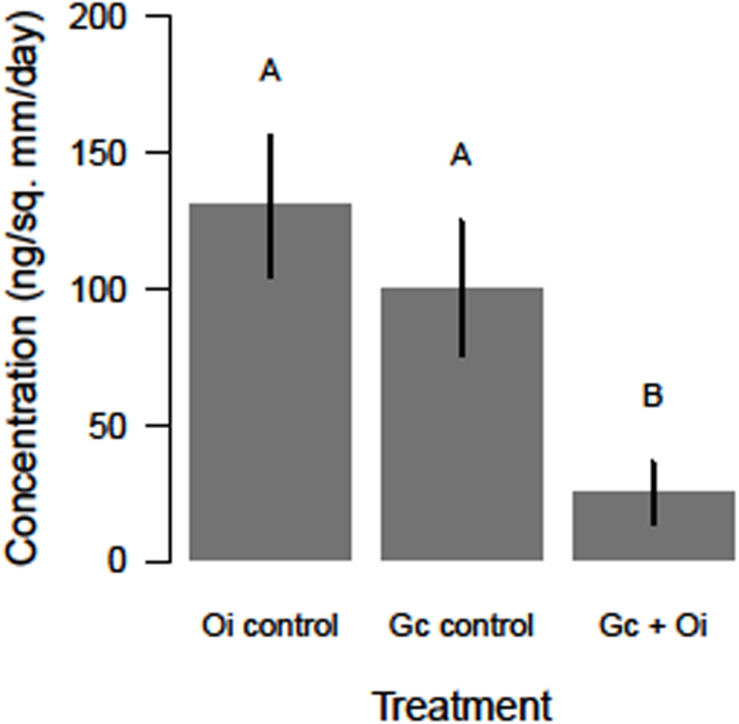
Differences in the total concentration (ng/mm^2^/day ± standard error) of volatile organic compounds detected in the headspace of *Ophiostoma ips* (Oi control) cultures, *Grosmannia clavigera* (Gc control) cultures, and these fungi together (Gc + Oi). Bars with different letter superscripts were different as indicated by Tukey’s Honest Significant Different tests.

The headspace VOCs detected from *G. clavigera* and *O. ips* exhibited quantitative variation. The total concentrations of VOCs the fungi emitted responded to the treatments, as a significant main effect of treatment was detected (*F*_(2,22)_ = 5.58, *P* = 0.011; Figure 2). Total FVOC concentrations from the combined treatment were 75 and 81% lower than those of the *G. clavigera* and *O. ips* controls, respectively. The fungal controls had similar concentrations of total FVOCs. Total FVOC concentrations did not vary by fungal isolate, as isolate main effects of isolate-treatment interactions were non-significant.

Emission of individual FVOCs also significantly varied among treatments (PERMANOVA *F*_(2,22)_ = 5.22, *P* = 0.011; Figure 1B), with the profiles of the combination treatment being significantly different from those of the *O. ips* control but similar to the *G. clavigera* control. Fungal isolate did not influence these profiles, as significant isolate main effects of isolate-treatment interactions were not detected. The lowest concentrations of individual FVOCs were consistently detected from the combination treatment, which had concentrations ranging from 92 to 34% lower than either of the fungal controls, depending on the compound (Table 2). However, the *O. ips* control tended to have the highest concentrations for most of the individual VOCs detected in that treatment.

**TABLE 2:** Mean concentration of individual and total fungal volatile organic compounds detected from headspace collections of *Grosmannia clavigera* (*N* = 10), *Ophiostoma ips* (*N* = 10), and *G. clavigera* plus *O. ips* grown on potato dextrose agar (*N* = 9).

	Mean concentration (ng mm^–2^ day^–1^; ±S.E.)		
	of compound		
	
Compounds	*Grosmannia clavigera*	*Ophiostoma ips*	*G. clavigera* + *O. ips*
Acetoin	2.16 (±0.78)	4.20 (±1.06)	0.73 (±0.45)
Ethyl acetate	2.03 (±0.68)	0.89 (±0.23)	0.49 (±0.23)
*cis*-Grandisol	0.30 (±0.15)	ND	0.20 (±0.11)
Isoamyl alcohol	33.87 (±8.55)	54.01 (±11.52)	9.37 (±4.59)
Isobutanol	48.74 (±12.70)	58.17 (±10.99)	11.13 (±4.68)
2-Methyl-1-butanol	8.05 (±2.31)	7.21 (±2.19)	1.57 (±0.76)
Phenethyl acetate	2.82 (±1.53)	ND	0.83 (±0.46)
Phenethyl alcohol	2.38 (±0.95)	5.93 (±2.02)	0.47 (±0.29)

*Compounds not detected in a given collection group are indicated with “ND.”*

### Fungal Growth Responses

**FIGURE 3:**
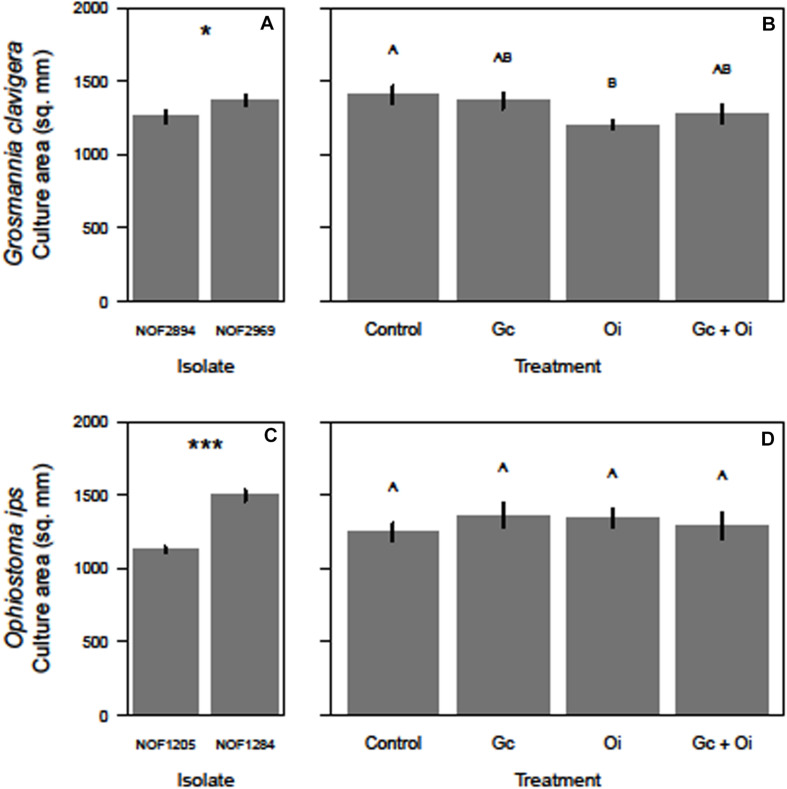
Differences in the growth (mm^2^) of the two isolates of *Grosmannia clavigera* (Gc) and *Ophiostoma ips* (Oi) when they grow alone (**A,C**, respectively) or in in a shared headspace environment (**B,D**, respectively). We used 4-day old cultures of each fungal species. Bars with different letter superscripts were different as indicated by Tukey’s Honest Significant Different tests. Statistical significance at *P* < 0.050 indicated by “*” and at *P* < 0.001 indicated by “***.”

The total growth (mm^2^) of *G. clavigera* and *O. ips* varied after the cultures were grown in the presence of VOCs from source fungal cultures, occurring either separately or in combination, or two PDA control plates (Figure 3).

For *G. clavigera*, the growth of response cultures significantly differed between the two isolates used (*F*_(1,32)_ = 4.58, *P* = 0.040; Figure 3A), with the growth of isolate NOF2969 was slightly (8%) larger than isolate NOF2894 after 3 days, independent of emission treatment (Figure 3A). However, the isolate of *G. clavigera* grown did not influence their response to the emission treatments, as there was no significant treatment-isolate interaction effect. The growth of response cultures was influenced by the emission treatments as a significant treatment main effect was detected (*F*_(3,32)_ = 3.43, *P* = 0.028; Figure 3B). Pairwise comparisons indicated that *G. clavigera* grew 15% less when exposed to VOCs of *O. ips* compared to when growing with controls (Figure 3B). However, *G. clavigera* growth was not influenced by VOCs from the *G. clavigera* or combination emission treatments (Figure 3B).

For *O. ips*, the growth of response cultures significantly differed between isolates (*F*_(1,32)_ = 104.38, *P* < 0.001), with the growth of isolate NOF1284 being 33% larger than that of NOF1205 (Figure 3C) after 4 days. However, the growth of *O. ips* was not influenced by the emission treatments (Figure 3D), as the main effect of treatment was not significant. The isolate-treatment interaction for *O. ips* response cultures was non-significant.

## Discussion

Fungal VOCs emitted by one ophiostomatoid fungus can be affected by the headspace of FVOCs from another species. The FVOC profiles from the combination treatment (*G. clavigera* and *O. ips* together) differed from those of the *O. ips* alone treatment but not from those of the *G. clavigera* alone. The contribution of *G. clavigera* to the FVOC profiles of the combination treatment does not necessarily explain the difference between profiles of the *O. ips* control and the combination treatments, as the incidence of detection and the concentrations of several compounds (e.g., acetoin, isoamyl alcohol, and phenethyl alcohol) in the combination treatment were lower than those in the *O. ips* control. Taken together, these findings suggest that the FVOCs emitted from some ophiostomatoid fungi can alter FVOC profiles of other fungal species. These results complement to those observed in other systems, where changes in FVOC profiles were elicited by physical (mycelial) interactions among multiple species of fungi sharing a niche (Hynes et al., 2007; El Ariebi et al., 2016). However, our findings suggest that such effects could also result from FVOC-mediated interactions between fungal species without physical contact. Currently, the mechanism underlying such inter-fungal interactions compare to those mediated by water soluble chemical signals secreted by fungi growing in close proximity is unknown (Hofstetter et al., 2005; Schmidt et al., 2015).

While FVOC profiles changed as a result of growing both *G. clavigera* and *O. ips* in a shared headspace environment, these changes did not influence the growth of interacting fungi. However, the growth of ophiostomatoid species can respond to FVOCs from other ophiostomatoid fungi, with the outcome of such treatments varying with the species of fungus. Hofstetter et al. (2005) showed that the growth rate of *Ophiostoma minus* was reduced by the volatiles emitted from actively growing *Entomocorticium* sp. *A* and *Ophiostoma ranaculosum* in the southern pine beetle (*Dendroctonus frontalis*) system. However, in the same study another fungus tested *Leptographium terebrantis* had no effect on *O. minus* growth rate. Similarly, Cale et al. (2016) showed that the outcomes of interactions among three ophiostomatoid fungi vectored by MPB can result in either no-effect or inhibition of fungal growth in response to their FVOCs. In particular, *G. clavigera* cultures growing in the headspace of FVOCs from cultures of *Ophiostoma montium* were 50% smaller than controls but the fungus *Leptographium longiclavatum* did not respond to FVOCs from *G. clavigera*.

The FVOC profiles emitted differed between *G. clavigera* and *O. ips*. While six compounds were common between species, two compounds were detected only when *G. clavigera* grew alone. The six FVOCs (acetoin, ethyl acetate, isoamyl acetate, isobutanol, 2-methyl-1-butanol, and phenethyl alcohol) have also been detected in headspace of several ophiostomatoid fungal species, likely because they are byproducts of primary metabolism during vegetative growth (Kandasamy et al., 2016; Cale et al., 2019). Furthermore, the similarities in FVOCs among different ophiostomatoid fungal species may also reflect a common ecological niche as fungi with a common ecological niche share similar FVOC profiles (Müller et al., 2013). While only detected from the headspace of *G. clavigera*, here, phenethyl acetate has been detected in FVOC profiles of some other ophiostomatoid fungi (e.g., *L. longiclavatum*, *Endoconidiophora polonica*) (Cale et al., 2016, 2019; Kandasamy et al., 2016). However, among ophiostomatoid fungi, *cis*-grandisol has only been reported from the FVOC profiles of *G. clavigera* (Cale et al., 2016, 2019), suggesting species-specific production of FVOCs.

There are a number of studies reported the ecological roles of many FVOCs including those ophiostomatoid species identified in this study, in other organisms including fungi, bark beetles, and other insect species (Davis et al., 2013; Kandasamy et al., 2016; Cale et al., 2019). Thus, we here will keep our discussion on the ecological roles of FOVs identified in this study short. Phenethyl alcohol can inhibit attraction of MPB and *D. frontalis* (Renwick et al., 1976; Pureswaran et al., 2000; Pureswaran and Borden, 2004; Sullivan et al., 2007). Here, *O. ips* emitted more phenylethyl alcohol than *G. clavigera*, suggesting that trees attacked by *I. pini* vectoring *O. ips* may be less attractive to MPB because of strong phenethyl alcohol emissions from the fungus. Such behavioral effects may reduce competition between MPB and *I. pini*. Conversely, isoamyl alcohol is emitted by a number of ophiostomatoid fungal species and attractive to *D. frontalis* (Brand et al., 1977). Although the behavioral responses of MPB and *I. pini* to isoamyl alcohol are unknown, the emission of this compound by *O. ips* and *G. clavigera* may suggest it plays similar roles in the chemical ecology of both beetles. 2-methyl-1-butanol is a common FVOC that attracts a wide diversity of insect species, representing several phyla (Davis et al., 2013; Zhao et al., 2015; Cale et al., 2016). Although only detected from *G. clavigera*, among reported FVOC surveys of ophiostomatoid fungi, *cis*-grandisol (grandlure I) is an important aggregation pheromone of several weevil species (Tewari et al., 2014).

Individual FVOCs may be accurately sampled using different methodologies. While, we collected FVOCs using an air entrainment approach, our recent study with the same fungal isolates extracted FVOCs from filtrate from liquid cultures growing in potato dextrose broth (Cale et al., 2019). Each of the eight compounds detected in the current study were also detected in the liquid culture. Furthermore, in both studies, phenethyl alcohol, isoamyl alcohol, 2-methyl-1-butanol, and isobutanol were determined to be dominant components of FVOCs, being detected in at least 90% of samples and were major components of total FVOC concentrations from *G. clavigera* or *O. ips* isolates growing alone. Similarly, the same individual FVOCs were determined, here, and by Cale et al. (2019) to be minor components of the FVOCs of the same fungi here. Specifically, acetoin was the compound least frequently detected and represented a small percentage of total FVOC concentrations. Furthermore, *cis*-grandisol and phenethyl acetate were detected only from cultures of *G. clavigera*, here and by Cale et al. (2019). However, it should be noted that the chemistry of fungal growth media can influence FVOC profiles, and should be chosen carefully when designing studies of FVOCs (Cale et al., 2019).

Caveats are an inherent part of conducting *in vitro* bioassays investigating fundamental aspects of ecological interactions. Several such caveats should be considered with the study, here. First, the two isolates of *G. clavigera* and *O. ips* used here differed in growth rate, which may not accurately reflect the range of growth rates of either species exhibit in nature or culture. However, this may not influence how representative the FVOCs detected are of the species as profiles of both isolates were qualitatively and quantitatively similar when standardized by culture area. Furthermore, such similarities in FVOC profiles may be further representative of a particular ecological function/niche (Müller et al., 2013). Second, FVOCs were collected from culture growing on artificial growth media. Substrate chemistry can influence FVOCs from at least ophiostomatoid fungi (Cale et al., 2019), and the FVOCs profiles determined here may differ from those emitted by the fungi growing on their natural substrate of pine vascular tissue. However, the use of artificial media is integral in fungal ecology as growing conditions of cryptic species either cannot be replicated under laboratory conditions or cannot be designed using natural substrates in a manner that affords a mechanistic understanding of the interactions under study (Crowther et al., 2018). For studies of FVOCs from fungal cultures on any growth media, sampling FVOCs from a non-inoculated control is critical to distinguishing between fungus- and media-emitted compounds. Lastly, in nature, *G. clavigera* and *O. montium* would interact under pine bark, an anaerobic environment when intact that is rich with plant primary and secondary metabolites (Hofstetter et al., 2005). The outcome of inter-fungal interactions in such an environment may differ from those shown here as substrate chemistry can at least affect the FVOC emissions from these fungi (Cale et al., 2019).

## Data Availability Statement

The raw data supporting the conclusions of this article will be made available by the authors, without undue reservation.

## Author Contributions

FW, JAC, and NE conceived the research project and wrote the manuscript. FW designed and performed the studies. JAC and AH assisted in running chemical analyses. JAC helped FW data analyses. All authors provided editorial advice.

## Conflict of Interest

The authors declare that the research was conducted in the absence of any commercial or financial relationships that could be construed as a potential conflict of interest.

## Supplementary Material

FIGURE S1 Fungal volatile collection apparatus. This is a closed-air system consisting of a 473 mL glass jar fitted with a metal cap fitted with two pairs of brass spigots. Spigots serve as channels to allow constant airflow (475 mL min^–1^) into and out of the glass jar. The inlet channel is attached to 30.5 cm piece of Teflon tubing packed halfway down with activated carbon (800 mg; 6–14 mesh; held in place with glass wool) in order to purify air entering the glass jar. This tubing is attached to a stainless-steel gang-valve connected to the outlet spigot of a bellows vacuum/pressure pump. A 15 cm section of Teflon tubing is attached to the outlet channel and attached to an adsorbant trap consisting of a 7.5 cm piece of Teflon tubing packed with 150 mg of activated carbon (held in place with glass wool). This trap collects culture headspace volatiles carried in the air stream as it flows out of the collection chamber. The trap is attached to 8 cm long tube that joins the outlet channel to a gang-valve connected to the inlet spigot of the pump. A tight wrapping of Teflon tape is used at sites of tube and fitting connections in order to prevent outside air from entering and, thus, contaminating the system.
