# Supplementary figures and images for: Exposure to Fungal Volatiles Can Influence Volatile Emissions From Other Ophiostomatoid Fungi
Source: Front Microbiol. 2020 Sep 17;11:567462. doi: 10.3389/fmicb.2020.567462 (PMC7527408; doi:10.3389/fmicb.2020.567462)

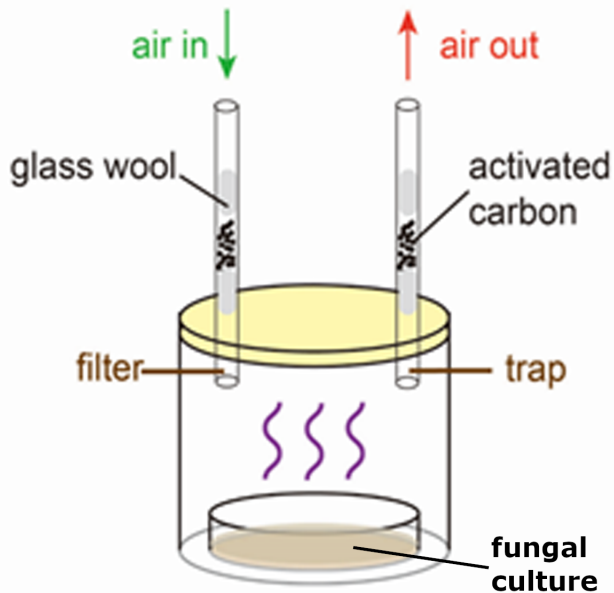

Supplement: FIGURE S1 — Fungal volatile collection apparatus. This is a closed-air system consisting of a 473 mL glass jar fitted with a metal cap fitted with two pairs of brass spigots. Spigots serve as channels to allow constant airflow (475 mL min–1) into and out of the glass jar. The inlet channel is attached to 30.5 cm piece of Teflon tubing packed halfway down with activated carbon (800 mg; 6–14 mesh; held in place with glass wool) in order to purify air entering the glass jar. This tubing is attached to a stainless-steel gang-valve connected to the outlet spigot of a bellows vacuum/pressure pump. A 15 cm section of Teflon tubing is attached to the outlet channel and attached to an adsorbant trap consisting of a 7.5 cm piece of Teflon tubing packed with 150 mg of activated carbon (held in place with glass wool). This trap collects culture headspace volatiles carried in the air stream as it flows out of the collection chamber. The trap is attached to 8 cm long tube that joins the outlet channel to a gang-valve connected to the inlet spigot of the pump. A tight wrapping of Teflon tape is used at sites of tube and fitting connections in order to prevent outside air from entering and, thus, contaminating the system. [file Data_Sheet_1.PDF]
